# Slow Scan Cyclic Voltammetry of Li-Ion Insertion in T‑Nb_2_O_5_ Reveals Hidden Peaks and Multi-Electron Redox

**DOI:** 10.1021/acselectrochem.5c00533

**Published:** 2026-03-19

**Authors:** Luke D. Salzer, Cami Christensen, Claire Gervais, Jacob D. Steeley, James R. Neilson, Justin B. Sambur

**Affiliations:** Department of Chemistry, 3447 Colorado State University, Fort Collins, Colorado 80523, United States

**Keywords:** energy storage, transition metal oxides, Li-ion insertion, structure-property relationships, pseudocapacitance

## Abstract

The orthorhombic phase of Nb_2_O_5_ formed at low temperatures (T-Nb_2_O_5_) is a promising alternative anode material for lithium-ion batteries due to its ability to reversibly (de)­lithiate at high rates without forming lithium metal, which is a major safety limitation of conventional graphite anodes. Despite decades of research, the cyclic voltammetry response of T-Nb_2_O_5_ remains poorly understood, with conflicting reports regarding the number and peak potentials of cathodic and anodic redox peaks in the voltammogram. While some studies report a single broad redox feature in cyclic voltammograms, others observe multiple peaks, yet often describe lithiation using a single overall reaction: T–Nb_2_O_5_ + *x*Li^+^ + *xe* ^–^↔T–Li_*x*_Nb_2_O_5_ for 0 < *x* < 2. For *x* = 2, Li insertion is accompanied by single-electron reduction of each Nb center (Nb^5+^ → Nb^4+^). In this work, we employ slow-scan cyclic voltammetry (SSCV) at an ultraslow rate of 1.5 μV/s to minimize resistive losses and kinetic limitations in the oxide. Under these near-equilibrium conditions, we achieve *x* = 3.0 in T-Li_*x*_Nb_2_O_5_ from 3.0–1.2 V, corresponding to more than one electron per Nb center, and resolve five distinct cathodic peaks during lithiation. Three broad peaks are assigned to structural transformations based on recent literature *in situ* synchrotron-based X-ray diffraction data [ HanH., . Nat. Mater. 2023, 22­(9), 1128–1135.37500959 10.1038/s41563-023-01612-2PMC10465368]: an orthorhombic-to-monoclinic distortion at *x* ≈ 0.5, followed by transitions to an amorphous insulating phase, and a tetragonal Li-rich layered rock salt structure at *x =* 2.0 and *x* = 2.5, respectively. In addition, two sharp peaks at 1.678 and 1.658 V, which merge into a single “super peak” at faster sweep rates (>100 μV/s), are attributed to surface-related electrochemical processes such as Nb reduction and Li-ion adsorption, surface phase transitions, or impurity B–Nb_2_O_5_. These features, widely observed in the literature but previously unassigned, underscore how electrode architecture (particles vs thin films), morphology, and scan rate conditions shape the voltammetric signature of T-Nb_2_O_5_. By employing ultraslow scan rates to suppress kinetic limitations and ohmic losses, we reveal previously hidden cathodic features in the cyclic voltammograms and directly correlate them with recently reported in situ structural phase transitions.

## Graphical abstract

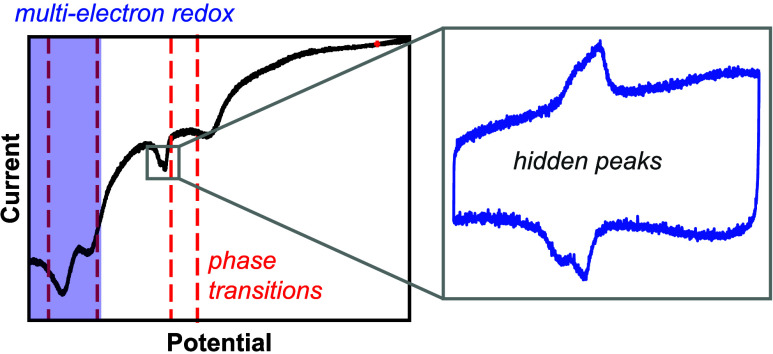

## Introduction

Nb_2_O_5_ first attracted attention as a cathode material for 2 V rechargeable batteries aimed at portable electronic applications.^1–3^ Kodama et al. claimed that, as early as 1998, monthly production of LiAl/Li_*x*_Nb_2_O_5_ secondary cells reached 500,000 units.^4^

Early fundamental studies on structural changes during Li-ion insertion in Nb_2_O_5_ used samples prepared by air annealing Nb metal at 500 °C. Samples prepared in this way form orthorhombic T-Nb_2_O_5_.^1,5^ Note, the nomenclature for Nb_2_O_5_ phases is confusing.^6^ Nb_2_O_5_ has several modifications that have been denoted as α, β, γ, ϵ, δ or LL­(TT), L­(T), B, M, H, N, P (or I, II, III).^5^ Brauer^7^ and Schäfer^5^ recommended L-Nb_2_O_5_ for the orthorhombic unit cell. The letter T denotes temperature (not the tetragonal phase). The orthorhombic phase forms at low temperature (e.g., heating Nb metal samples to 500 °C) and is denoted T-Nb_2_O_5_, where the T indicates the low formation temperature. Hence, L-Nb_2_O_5_ is equivalent to T-Nb_2_O_5_.^6^

Reichman and Bard investigated Li-ion insertion in T-Nb_2_O_5_ electrodes using LiClO_4_–propylene carbonate as the electrolyte and identified the formation of a new Li_*x*_Nb_2_O_5_ phase when *x* reached 0.4.^8^ They reported a capacity of 150 mAh/g and observed lithiation up to *x* = 1.5 at 1.0 V vs Li/Li^+^. Only modest shifts in the X-ray diffraction (XRD) patterns were observed with increasing lithiation, which they attributed to structural distortions within the Nb_2_O_5_ lattice. Nakajima and Watanabe were also among the first to investigate the structural evolution of Nb_2_O_5_ electrodes prepared in the same way using ex situ X-ray photoelectron spectroscopy (XPS) and X-ray diffraction (XRD).^2^ When cycled down to 1.0 V at current densities of 0.1–0.2 mA/cm^2^, the electrodes exhibited capacity loss and evolving charge–discharge profiles over repeated cycles. In contrast, stable cycling over 1000 cycles was achieved by restricting the voltage window to 2.5–1.5 V at a current density of 0.2 mA/cm^2^. A particularly notable observation was the monotonic decrease in the Nb:O atomic ratio from 0.5 to nearly zero at *x* = 0.5, suggesting that lattice oxygen migrates to the surface upon lithiation. The authors attributed the lithiation process to the formation of a disordered Li_*x*_Nb_2_O_5_ phase with a disrupted Nb–O framework. The Nb:O ratio trend is somewhat curious, as it is not directly reflected in the nominal redox reaction: T – Nb_2_O_5_ + *x*Li^+^ + *xe* ^–^↔T – Li_*x*_Nb_2_O_5_ (where *x* ≥ 2), highlighting the possibility that additional redox processes or structural rearrangements contribute to the observed electrochemical behavior.

Kumagai and Tanno initially examined the charge–discharge behavior and cycle stability of T-, B-, N-, and H-phases of Nb_2_O_5_ that could be accessed via air annealing of Nb metal at different temperatures.^1^ The authors showed that T-, B-, N-, and H-phases exhibit distinct cycling behavior, with the H-phase exhibiting the greatest capacity (240 mAh/g) upon cycling to 1.0 V during discharge. T-Nb_2_O_5_ electrodes exhibited poor cycling stability upon cycling to 1.0 V and lower capacity in LiBF_4_ electrolyte compared to LiClO_4_ electrolyte. Impressively, they reported stable cycling over a one year period (1000 cycles) at 20 mAh/g for the discharge potential range of 2.2 to 1.5 V and the charge potential range of 1.5 to 3.0 V. Kumagai also reported <30 mA/g capacity for Na-ion insertion in T-Nb_2_O_5_ relative to 200 mA/g for Li^+^, as well as poor cycle stability upon cycling to 1.0 V vs Li/Li^+^. The authors analyzed XRD diffraction patterns and concluded that the crystallite size decreases upon inserting Li-ions into the lattice. The Li-ion diffusion coefficient increased from *x* = 0.2 to 0.8 and then leveled off until *x* = 2.0.

In 1987, Ohzuku et al. reported an intriguing result: T-Nb_2_O_5_ electrodes cycled without carbon binder at a low rate (∼0.025C) accommodated up to three Li ions per formula unit (*x* = 3).^6^ The cell voltage stayed roughly constant at approximately 1.0 V as the material was lithiated from 2 < *x* < 3. Based on ex situ XRD data, where diffraction peaks broadened and shifted slightly to lower angles, the authors concluded that the electrochemical reduction of T-Nb_2_O_5_ in Li-ion electrolytes proceeds via a topotactic reaction (i.e., a process in which ions are inserted while the overall framework of the host crystal is largely preserved). In contrast to the two-phase process proposed by Reichman and Bard, Ohzuku et al. noted that their proposed single-phase (topotactic) mechanism is likely due to differences in the phase purity and type of the starting material. The authors developed analytical expressions to model the charge–discharge behavior and proposed the following reaction: Nb_2_^5+^O_5_^2–^ + *x*Li^+^ + *xe* ^–^↔Li_*x*_Nb_2 – *x*_^5+^Nb_*x*_^4+^O_5_^2–^ for 0 < *x* < 2, where the Nb^5+/4+^ ions are located in 1­(a) and 1­(f) sites in the lattice. This work represents one of the earliest demonstrations of multi-electron reduction in T-Nb_2_O_5_ and analytical expressions to quantify the S-shaped charge–discharge profile of T-Nb_2_O_5_.

Kodama et al. investigated the structural evolution of various Nb_2_O_5_ phases, including tetragonal (denoted with the lower case letter t-, whereas upper case T- denotes temperature) and orthorhombic polymorphs, during lithium intercalation using both in situ XRD and X-ray absorption fine structure (XAFS) techniques.^4^ Cells were cycled to 1.2 V vs Li/Li^+^ in 1 M LiPF_6_ in ethylene carbonate electrolyte. The discharge curves exhibited sloping voltage profiles that decreased monotonically with increasing capacity, and the authors attributed the entire electrochemical process to a single redox reaction. They reported a high discharge capacity of approximately 230 mAh/g and a recharge capacity of around 190 mAh/g. Notably, the tetragonal phase sample synthesized at 1000 °C contained a minor monoclinic Nb_2_O_5_ phase, which exhibited excellent cycling stability (over 1000 cycles within the 2.2–1.5 V window). In situ XRD showed minimal unit cell expansion upon lithiation, while in situ XAFS confirmed a single-electron Nb^5+^ → Nb^4+^ redox process. However, the exact fraction of the monoclinic phase was not quantified, and its influence on the lithiation process and associated structural changes remains unclear.

Kim et al. further explored the phase-dependent electrochemical properties of Nb_2_O_5_ and synthesized amorphous, TT-, and T-phase Nb_2_O_5_ nanoparticles via sol–gel routes and tested the materials as pure powder electrodes without binders or conductive additives.^9^ T-Nb_2_O_5_ showed the best performance for fast charging applications, delivering nearly 400 F g^–1^ reversibly within 12 seconds, which is among the fastest responses reported for transition-metal oxides. Cyclic voltammograms (CVs) measured at 10 mV/s showed two prominent cathodic peaks at 1.75 and 1.5 V. High resolution transmission electron microscopy (TEM) confirmed formation of a solid–electrolyte interphase. Strikingly, thin-film T-Nb_2_O_5_ electrodes showed markedly different voltammetry: the two peaks collapsed into a single broad feature at 10 mV s^–1^, and at slower sweep rates a sharp redox couple emerged at 1.9 V along with a broad, poorly defined feature near 1.5 V.^10,11^ Multiple cathodic peaks do not appear in electrochemically deposited T-Nb_2_O_5_ films.^12^ Thus, particle-based and thin-film electrodes yield qualitatively different electrochemical signatures, underscoring the importance of material morphology and surface atom-to-bulk atom ratio in interpreting T-Nb_2_O_5_ redox behavior.

Since the influential 2013 report by Augustyn and Dunn on intercalation pseudocapacitance in T-Nb_2_O_5_,^10^ much attention has centered on kinetic power-law analysis of peak current versus sweep rate, with less emphasis on the number, position, and origin of CV peaks. This work focuses on clarifying the cyclic voltammetry response of particle-based T-Nb_2_O_5_ in Li-ion electrolytes. By employing extremely slow scan rates, we approach near-equilibrium conditions and minimize ohmic losses. Here, using slow-scan CV at 1.5 μV s^–1^, we resolve three broad cathodic peaks during lithiation of T-Nb_2_O_5_. The peak potentials align with structural transformations recently observed in single-crystalline T-Nb_2_O_5_.^13^ Over the potential window 3.0–1.2 V, we achieve *x* = 3 in T-Li*_x_* Nb_2_O_5_, consistent with Ohzuku et al.,^6^ and we propose specific electrochemical reactions to account for the observed CV features.

## Experimental Methods

### Synthesis of T-Nb_2_O_5_

The samples were synthesized via high temperature solid state synthesis reactions following previous literature.^14^ NbO_2_ (Alfa Aesar, 99%) powder was ground in an agate mortar and pestle and then pressed into pellets using a hydraulic press (Caver Model: 4350.L) using 2 tons of pressure. The pellets were placed in an alumina crucible and platinum boat and heated open to air in a Thermo Scientific Lindberg Blue M tube furnace. The samples were heated from 25 °C at a rate of 10°C/min to 525 °C, held for 100 h, and allowed to cool naturally in the oven, before the pellet was ground again for approximately 5 min prior to characterization.

### Sample Characterization

The samples were characterized by powder X-ray diffraction (XRD) using a Bruker D8 Discover DaVinci-Powder Diffractometer (Cu Kα radiation). To do so, the metal oxide powders were suspended in ethanol and drop-casted onto a Si B-doped zero diffraction slide. Rietveld refinement was performed using Bruker’s Diffrac.Topas software using CIF files from Inorganic Crystal Structure Database (ICSD CC-1840).

### Half-Cell Construction

Coin cell batteries were constructed in an argon glove box using the following components: stainless steel cases (MTI Corp, CR2032), a stainless-steel wave spring (MTI Corp, CR20WS), a stainless-steel spacer (MTI Corp, CR20-Spacer-05), and a glass microfiber (VWR, 691) separator. The metal oxide composite electrode was prepared by combining T-Nb_2_O_5_ particles, conductive carbon (Super P, Alfa Aesar), and poly­(vinylidene fluoride) binder (PVDF, Sigma-Aldrich) in a 5:4:1 ratio by mass and grinding the mixture using an agate mortar and pestle. This mixture was dispersed in *N*-methyl pyrrolidone (NMP, Sigma-Aldrich) until the solution was slightly viscous. The slurry was doctor-bladed onto a copper substrate to cast a 100 μm-thick film. The film was dried overnight in vacuum at approximately 120°C. The dried film was punched into several 1/4 inch diameter electrodes and transferred into an argon glove box. The metal oxide composite electrode served as the cathode and lithium metal served as the anode. Coin cells were constructed with the MTI coin cell case using a 1/2 inch diameter lithium metal electrode, and a 5/8 inch diameter separator was laid on top. We compared two electrolyte systems in this work. To do so, 80.0 μL of either 1 M LiClO_4_ in propylene carbonate (PC, Sigma-Aldrich) or 1 M LiPF_6_ dissolved in a 1:1 volume ratio of ethylene carbonate/dimethyl carbonate (EC/DMC, Sigma-Aldrich) was pipetted onto the separator. The cathode was placed on the separator after it had become saturated with electrolyte, followed by the stainless-steel spacer, spring, and finally the end-cap. The cells were pressed with 0.9 tons of pressure using a compact digital pressure controlled electric crimper (MTI MSK-160E). The cells rested for 12 h before electrochemical measurements to allow the electrolyte to saturate the separator.

### Electrochemical Measurements

The coin cells were cycled on an Arbin battery tester (LBT-20084) at a C-rate of 1 C or C/3. The C-rate was calculated according to *I* = (*Q*_theoretical_ × *m*)/*t*, where *I* is the current applied, *m* is the mass of active material, and *t* is time in hours. The theoretical capacity (202 mAh/g) is calculated according to *Q*_theoretical_ = *nF*/3.6*M*, where *n* is the number of electrons transferred per formula unit (here we used *n* = 1), *F* is Faraday’s constant, 3.6 is a conversion factor between coulombs and mAh/g, and *M* is the molecular weight. Following constant current cycling, SSCV measurements were conducted on a CH instrument (CHI 1240 and CHI 1010A) and Autolab potentiostat (PGSTAT128N).

## Results

We synthesized T-Nb_2_O_5_ particles by heating NbO_2_ in air at 525 °C for 100 h (see Experimental Methods section for details) and investigated the electrochemical properties using galvanostatic charge/discharge measurements and SSCV.

Figure 1 shows the powder X-ray diffraction (PXRD) pattern of the synthesized material together with Rietveld refinement. The refinement was performed using crystallographic information for T-Nb_2_O_5_ (Pbam space group, ICSD collection code 1840) as the primary phase. While the overall diffraction pattern is dominated by reflections consistent with T-Nb_2_O_5_, minor deviations between the calculated and observed peak positions highlight known limitations of available structural models for this phase. Careful examination of the diffraction data reveals a small contribution from the B-Nb_2_O_5_ polymorph, which is known to form under similar solid-state synthesis conditions.^5,14^ In contrast, NbO_2_ does not produce discernible diffraction features within the sensitivity of the measurement and, if present, must be below the detection limit of laboratory PXRD. Due to the imperfect fit of the primary T-Nb_2_O_5_ phase, precise quantification of impurity phases is inherently uncertain; however, conservative refinement places an upper bound of ∼2 wt % on the B-Nb_2_O_5_ content (Table S1). The potential contribution of a minor B-Nb_2_O_5_ phase to the observed SSCV response is discussed further below.

**Figure 1:**
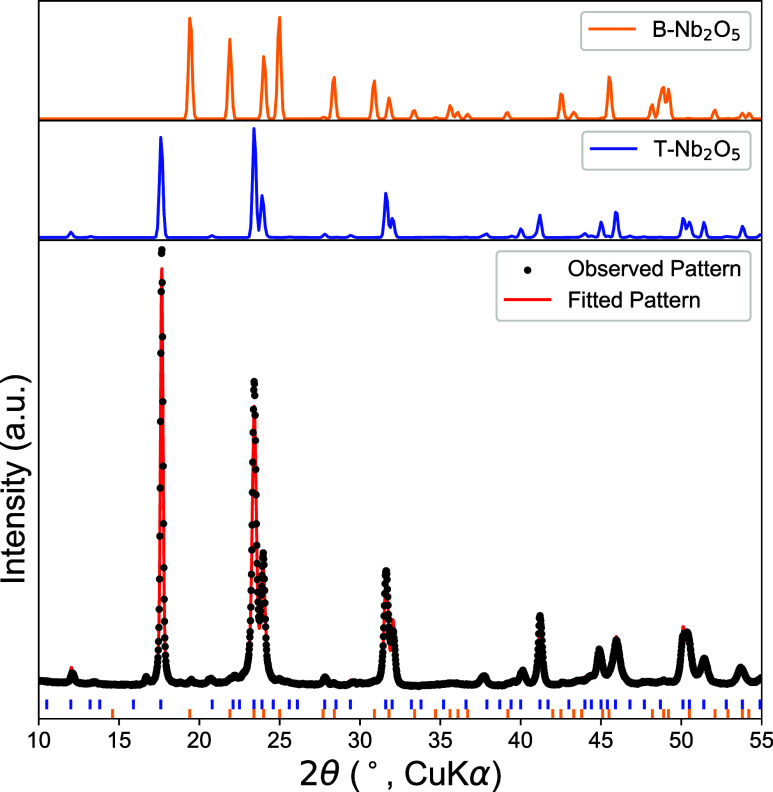
(Top) Diffraction pattern of NbO_2_ (green trace), T-Nb_2_O_5_ (blue trace, ICSD collection code 1840), and B-Nb_2_O_5_ (orange trace). (bottom) PXRD pattern of the reaction products obtained by heating NbO_2_ in air at 525°C for 100 h. The black dots represent the PXRD data and the red line represents the Rietveld refinement result using T-Nb_2_O_5_ alone (see Table S1 for refinement details and results).

Having characterized the structure of the reaction products, we investigated the electrochemical behavior of the material in Li-ion coin cells. We prepared composite electrodes with a similar metal oxide-to-carbon ratio to minimize ohmic losses;^15–17^ we focused on the fundamental voltametric behavior rather than battery performance. First, we evaluated the cycle stability of these T-Nb_2_O_5_ electrodes using slow galvanostatic cycling in different electrolytes and for two different cutoff voltages. Figure 2a,b shows the charge–discharge curves for T-Nb_2_O_5_ in 1 M LiClO_4_ in PC using 1.2 and 1.0 V vs Li/Li^+^ cutoff voltages, respectively. During first discharge (i.e., lithiation) to 1.2 V, the cell achieves a maximum capacity of 217 mAh/g, which is greater than the 202 mAh/g theoretical capacity (assuming 1 electron per Nb atom). On the next cycle, the capacity decreases to 172 mAh/g, either due to Li-ion trapping in the metal oxide, metal oxide particle detachment from the composite electrode, and/or reactions with the electrolyte. The remaining charge–discharge cycles are qualitatively similar, indicating the electrode materials remain intact and stable upon repetitive cycling in 1 M LiClO_4_ using a cutoff voltage of 1.2 V. We observed poor cycle stability using a 1.0 V cut off voltage (Figure 2b). Under these conditions, we observe a large initial discharge capacity of 251 mAh/g, followed by a large capacity loss after cycle 1 and a monotonic capacity loss with additional cycling. The large initial discharge capacity can be attributed to multi-electron redox (i.e., Nb^5+^ reduction to Nb^3+^) as well as possible electrolyte decomposition at voltages closer to 1 V vs Li/Li^+^.^18^ In 1 M LiPF_6_, we still observe an initial capacity loss after 1 cycle using a 1.2 V cutoff voltage (Figure 2c). There is some indication the cell stability improves in LiPF_6_, as indicated by overall superior capacity retention >150 mAh/g over 20 cycles. Yet, we still observe poor cycle stability in LiPF_6_ upon extending the cutoff voltage to 1.0 V (Figure 2d). An electrochemical signature of cycle instability appears in the d*Q*/d*V* plots (Figure 2f,h). Upon cycling the cells to 1.0 V, a new feature appears at positive potentials (2.1 V) that does not appear upon cycling the cells to 1.2 V. This signature appears after the fourth cycle and then disappears with additional cycling and is associated with long term electrochemical irreversibility. A key point of Figure 2 is that the cutoff voltage affects cycle stability more so than the electrolyte composition. This result aligns with findings by Kumagai et al.,^3^ who reported consistent charge–discharge behavior across various solvents, including LiClO_4_ in propylene carbonate, sulfolane, butyrolactone, and dimethyl sulfoxide.

**Figure 2:**
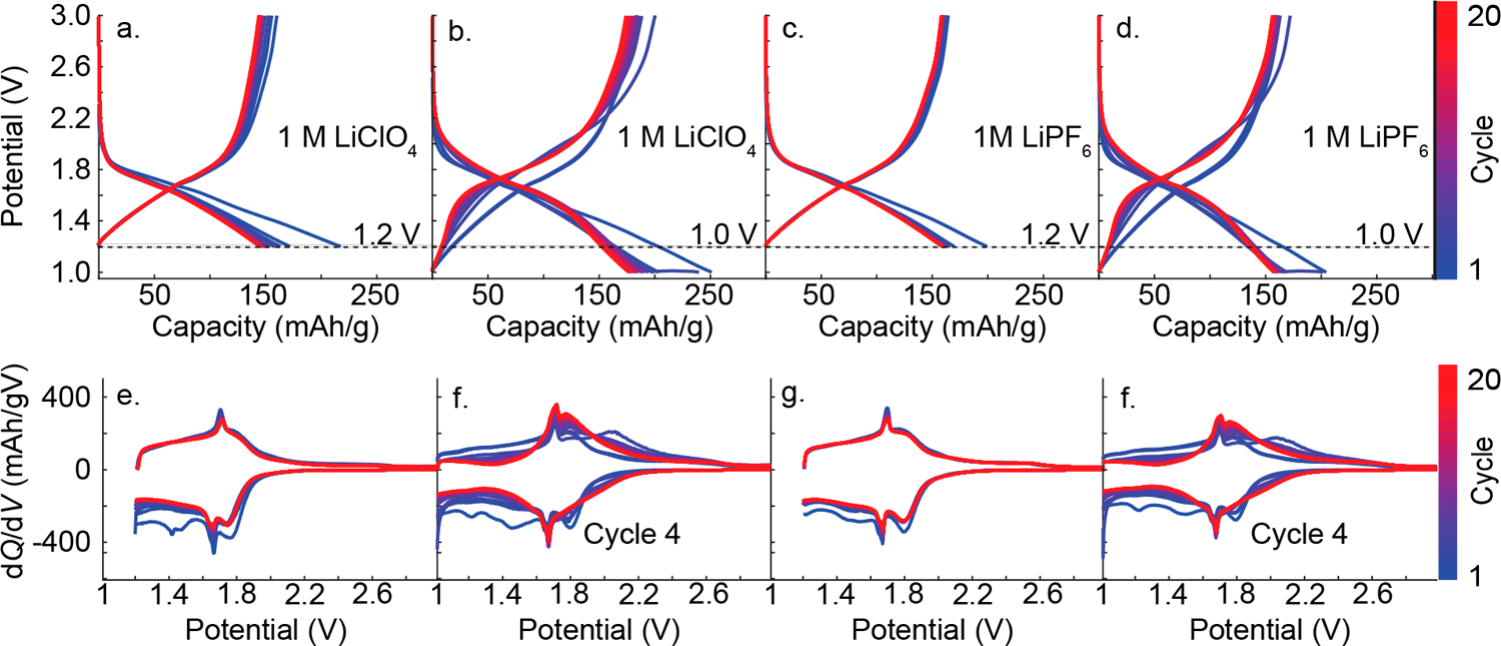
(a, b) Charge–discharge curves at C/*3*-rate using 1 M LiClO_4_ in PC and (c, d) 1 M LiPF_6_ in a 1:1 ratio of EC/DMC. The horizontal dashed line denotes the 1.2 V cutoff voltage used in panels a and c. (e, f) d*Q*/d*V* plots for 1 M LiClO_4_ in PC and (g, h) 1 M LiPF_6_ in a 1:1 ratio of EC/DMC. The positive d*Q*/d*V* feature at 2.1 V occurs after cycle 4, as indicated in panels f and h.

Having established stable cycling conditions for these T-Nb_2_O_5_ electrodes, we performed SSCV experiments in the plateau region of Figure 2a (1.85 to 1.55 V), where charge–discharge behavior is cycle independent and redox waves appear in CV data. Figure 3a shows SSCV data over the scan rate (*v*) range of 1.5 to 400 μV/s for a T-Nb_2_O_5_ electrode that had undergone 20 charge–discharge cycles from 3.0 to 1.2 V in 1 M LiClO_4_ in PC. Note, a single scan at 1.5 μV/s over a 300 mV potential window requires approximately 4.6 days to complete the measurement. The cell current expectedly increases with *v* and reduction and oxidation peaks appear within the potential range of 1.55 to 1.75 V. At the slowest sweep rates, the current is likely not limited by resistance, interfacial charge transfer kinetics, or mass transport. Figure 3b shows normalized CV data to better visualize the redox peak evolution as a function of *v*. The electrochemical behavior in Figure 3 appears in multiple coin cells (Figures S1 and S2). Figure S4 shows non-normalized 1.5–10 μV/s data. The normalized CVs were first smoothed using a 5-point moving average and subsequently normalized to the maximum cathodic current during the cathodic scan at each scan rate. At 1.5 μV/s, a pair of reduction peaks appear at *E*_p,c1_ = 1.678 V and *E*_p,c2_ = 1.658 V, respectively. We refer to the reduction peak currents and potentials as *i*_p,c1_, *E*_p,c1_, *i*_p,c2_ and *E*_p,c2_. Corresponding oxidation waves appear at *E*_p,a2_ = 1.673 V and *E*_p,a1_ = 1.693 V, yielding a peak splitting of Δ*E*_p,1_ = 15 mV and Δ*E*_p,2_ = 15 mV, respectively. The appearance of sharp, well-defined peaks at very slow scan rates is a known intrinsic feature of T-Nb_2_O_5_ and is not attributable to conductive carbon. Prior studies have reported similar peak shapes in electrodes containing no added carbon (Supporting Figure 3 of ref 10). Griffith et al.^14^ reported sharp peak features in d*Q*/d*V* plots upon lithiating T-Nb_2_O_5_ particle electrodes with low- and high-carbon content. Together, these studies demonstrate that the peaks originate from the intrinsic redox behavior of T-Nb_2_O_5_, with their prominence likely modulated by particle morphology, electrode design, and rate/cycling conditions rather than by carbon content. Interestingly, the peaks do not appear in single-crystal thin film electrodes,^13^ suggesting the peaks stem from the high number of surface sites in particle-based electrodes. Although a relatively high carbon content was employed to minimize resistive limitations at ultra-slow scan rates, similar Li insertion levels and sharp cathodic features have been reported for carbon-free T-Nb_2_O_5_ electrodes,^10,11^ indicating that these features are intrinsic to the active material. The Discussion section analyzes the total charge associated with the sharp peaks and compares it to the total number of Nb atoms in the sample. The integrated charge corresponds to less than 2% of the Nb atoms, or approximately 0.4% of all atoms in the sample.

**Figure 3:**
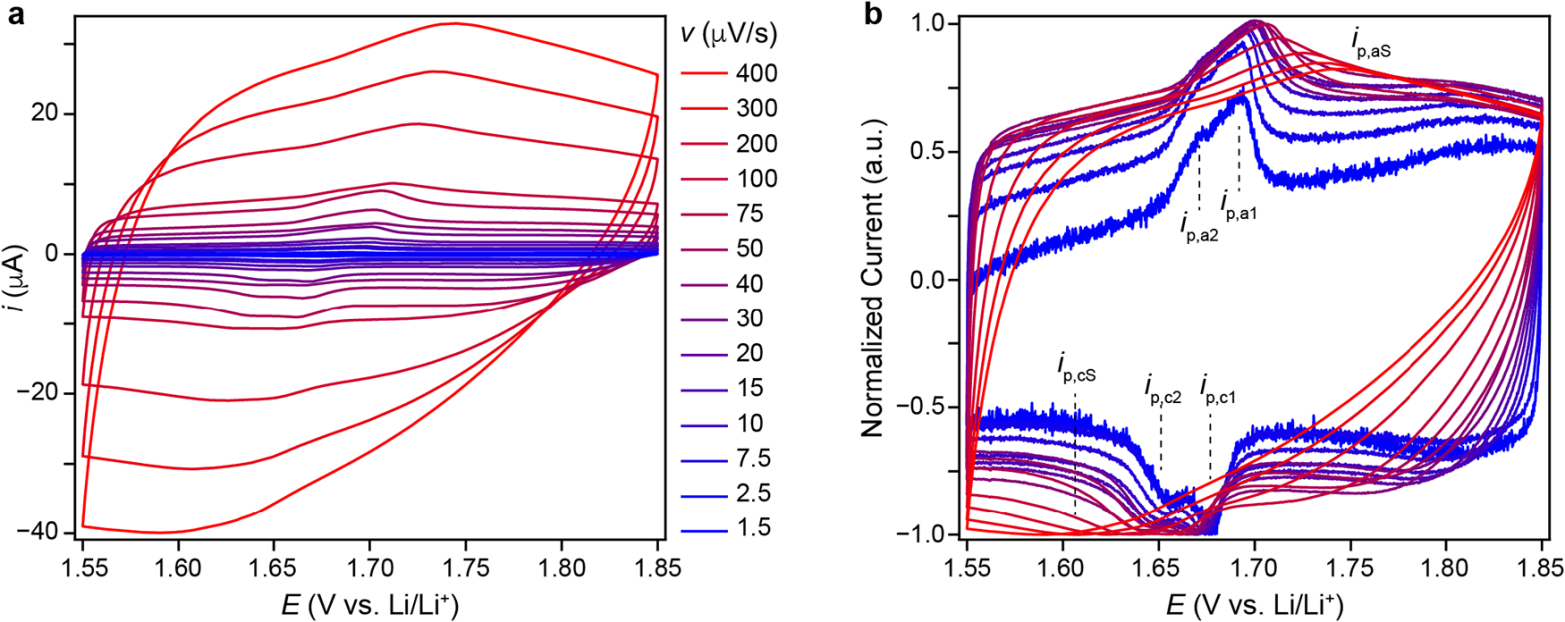
(a) SSCV of T-Nb_2_O_5_ electrode that underwent 20 charge–discharge cycles from 3.0 to 1.2 V in 1 M LiClO_4_ in PC. (b) Same data as in panel (a) normalized with respect to the maximum cathodic current.

As the scan-rate increases, the reduction (oxidation) peaks shift to more negative (positive) potentials, and the peaks become less prominent as the background current increases. At 100 μV/s and faster scan rates, the two reduction redox waves merge into a single “super peak” that is commonly reported in the literature.^4,11–14,18–23^ We refer to the cathodic and anodic “super peak” currents and potentials in Figure 3 as *i*_p,cS_,*E*_p,cS_ and *i*_p,aS_,*E*_p,aS_, respectively. To the best of our knowledge, this is the first report of the distinct reduction peaks at 1.678 and 1.658 V coalescing into a single “super peak” at faster scan rates.

Next, we analyzed how the peak currents and potentials in Figure 3 scale with *v*. The scan-rate dependence was evaluated using the peak current extracted directly from the CVs, without background subtraction. Figure 4 shows log *i*_p_-log *v* plots for the different cathodic and anodic CV peaks from the data in Figure 3a. *i*_p,a1_ and *i*_p,a2_ scale linearly with *v* for sweep rates 1.5 to 400 μV/s (the slope values are 1.0 in Figure 4a). *i*_p,aS_ also scales linearly with *v*, but with a smaller slope value of 0.82. We observed similar trends for the cathodic peak currents (Figure 4b).

**Figure 4:**
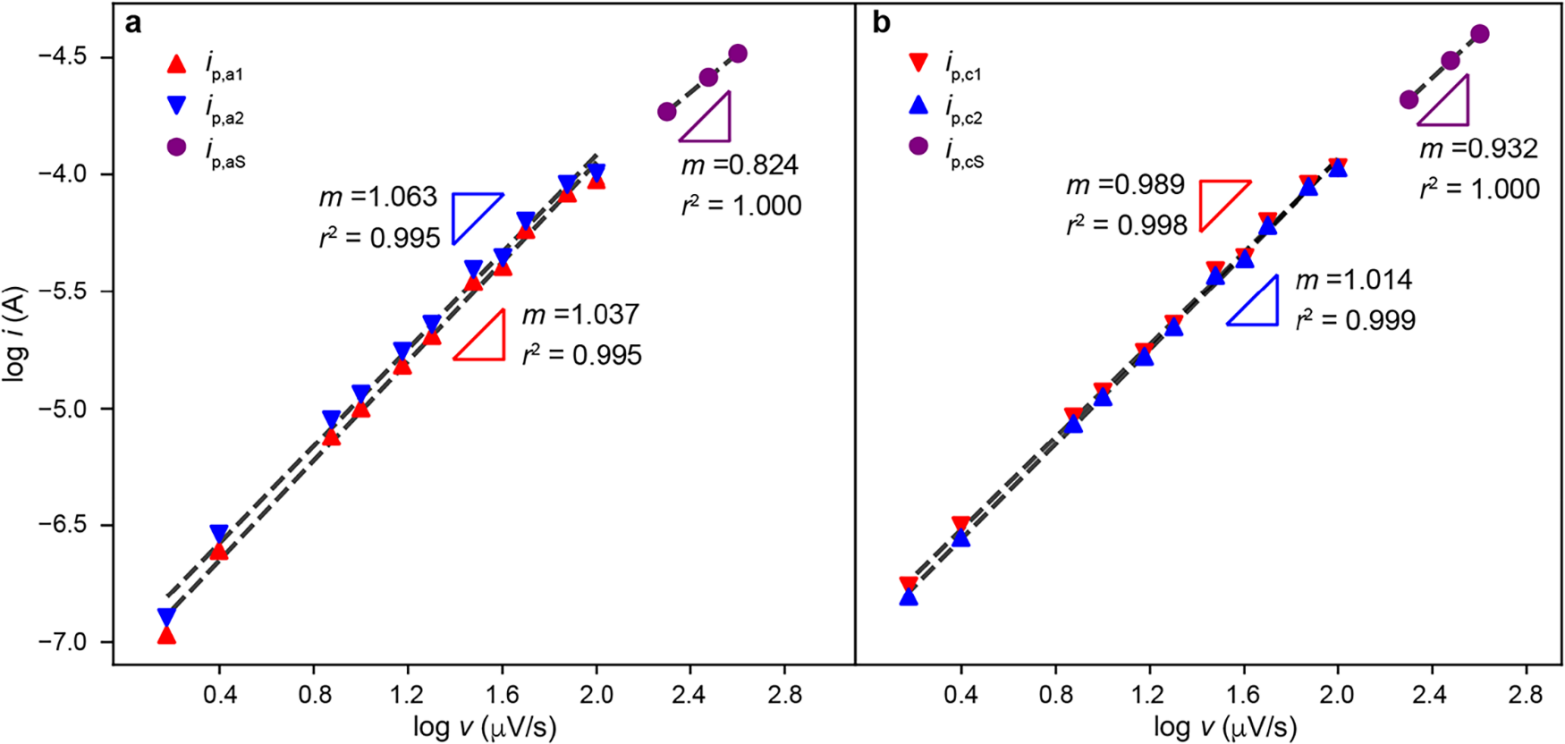
log *i*_p_-log *v* plots for (a) anodic peaks *i*_p,a1_, *i*_p,a2_, and *i*_p,aS_ defined in Figure 3. (b) Same as (a) but for the cathodic peaks in Figure 3.

Figure 5 plots the peak separation values versus *v*. The cathodic (anodic) peak potentials monotonically shift to more negative (positive) potentials with increasing sweep rate. At 1.5 μV/s, the peak separation values for peak 1 and peak 2 are both 15 mV. These peak separation values are smaller than 59 mV, corresponding to the expected peak separation for a one-electron, electrochemically reversible redox reaction under mass transport-limited conditions. Instead, the small peak separation values are consistent with an electrochemical process that is not limited by mass transport, such as the electrochemical reduction of Nb surface atoms and adsorption of Li-ions,^24^ a surface phase transition^25–27^ (such as a change in the coordination sphere of oxygens around Nb centers from NbO_6_ to NbO_7_ and NbO_8_ coordination),^28^ or surface reactions with the electrolyte.^9^ We discuss the origin of the sharp peaks in Figure 3b further below. At 200 μV/s, corresponding to the slowest sweep rate at which the “super peak” appears, the peak separation is 96 mV.

**Figure 5:**
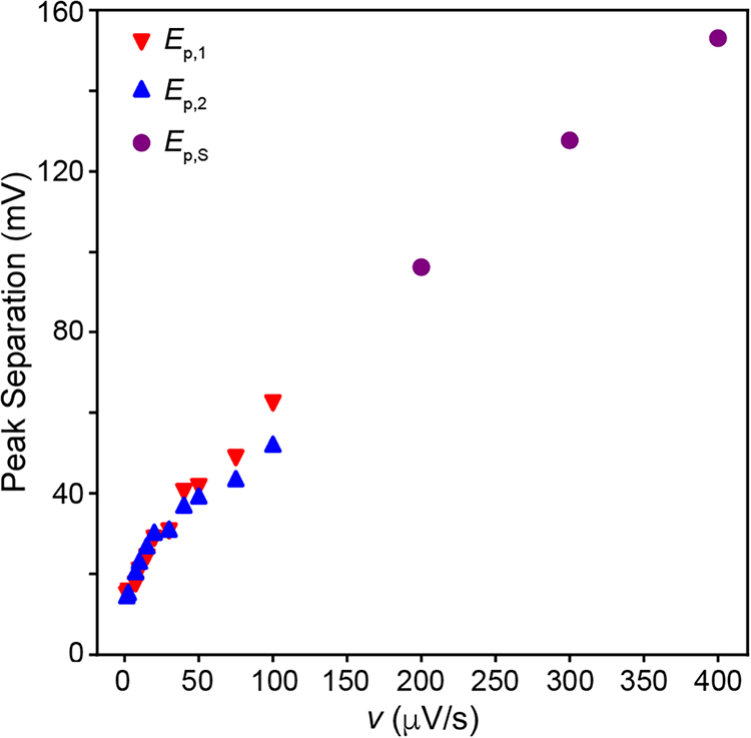
Anodic and cathodic peak separation values versus v from the SSCV data in Figure 3.

Finally, we investigated the full electrochemical lithiation behavior of T-Nb_2_O_5_ using slow-scan cyclic voltammetry (SSCV) from 3.0 to 1.0 V at a scan rate of 1.5 μV/s. Each sweep took over 15 days to complete. To manage this practically, we conducted the experiment in two stages: first, we performed a linear sweep from 3.0 to 1.0 V and saved the data at the midpoint (1.0 V). We then initiated a separate sweep in the reverse direction, from 1.0 V back to 3.0 V. As a result, the data in Figure 6 are presented as two separate sweeps. For the first 148 h, we observed anodic currents from 3.0 to 2.2 V, possibly due to de-lithiation of trapped Li-ions from previous cycles. At 2.56 V, cathodic currents flow (also see Figure 7). Hence, for pre-cycled electrodes, applying potentials more positive than 2.56 V fully oxidizes the T-Nb_2_O_5_ material and electrochemical reduction and lithiation of T-Nb_2_O_5_ commences at 2.56 V. The cathodic current steadily increases from 2.56 V until the first prominent cathodic reduction wave appears at 1.95 V. After that peak, the current decreases slightly with increasing negative potentials until the same two sharp cathodic peaks appear at 1.678 and 1.658 V (previously shown in Figure 3b). As the potential moves more negative than 1.6 V, the current steadily increases again until two more broad cathodic peaks appear at 1.35 and 1.24 V. Finally, a dramatic current increase takes place at 1.08 V. Upon reversing the scan direction at 1.0 V, we did not observe oxidation waves for any of the cathodic peaks observed over the potential range of 1.2 to 1.8 V despite observing reproducible reduction and oxidation peaks when a narrow potential scan range was employed in Figure 3. The event responsible for the large cathodic current increase at 1.1 V is likely responsible for the irreversible electrochemical behavior on the reverse scan, consistent with the cycling data in Figure 2. The irreversible behavior could be due to delamination of T-Nb_2_O_5_ from the current collector as well as Nb loss at the solid/electrolyte interface.^28^ An oxidation wave appeared at more positive potentials (1.92 V), yielding a peak splitting of 30 mV for the first redox wave with an *E*_1/2_ value of 1.935 V.

**Figure 6:**
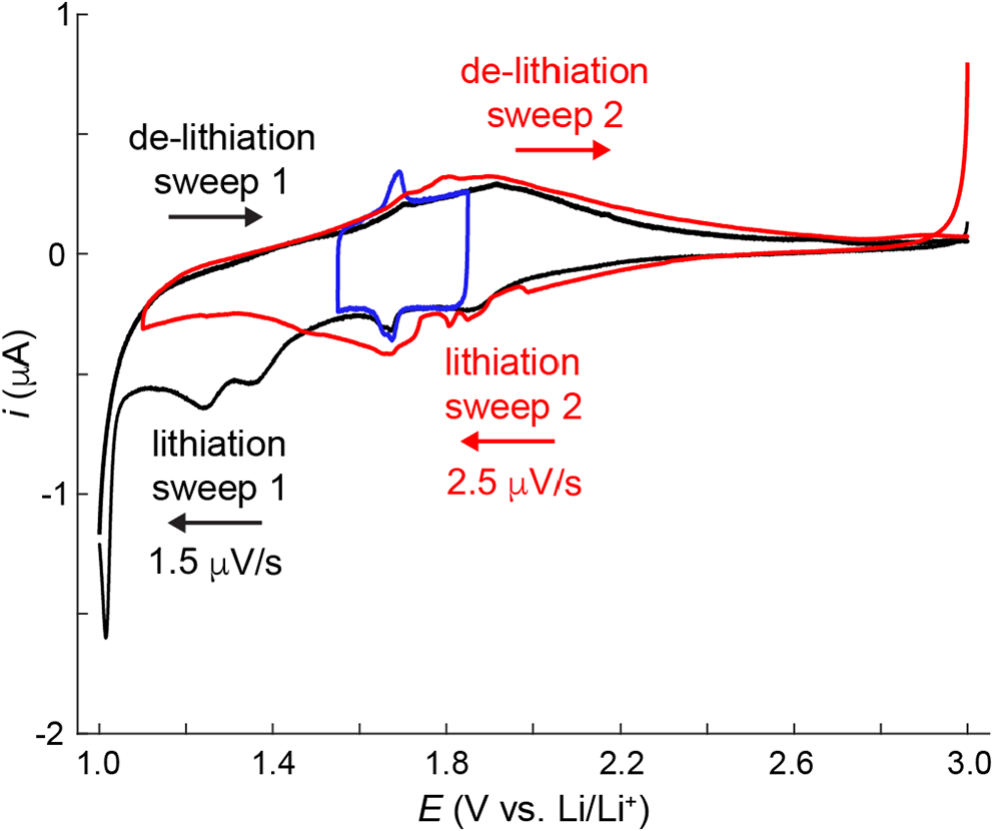
SSCV data obtained from the same T-Nb_2_O_5_ electrode as shown in Figure 3. This electrode underwent 20 galvanostatic charge/discharge cycles and repeated cycling over the narrow potential range, as shown in Figure 3. The blue trace shows the 1.5 μV/s CV data from Figure 3a. The black line represents the first sweep from 3.0 to 1.0 V at 1.5 μV/s. The red line represents the second CV sweep obtained at 2.5 μV/s. The blue line represents the 1.5 μV/s data from Figure 3.

**Figure 7:**
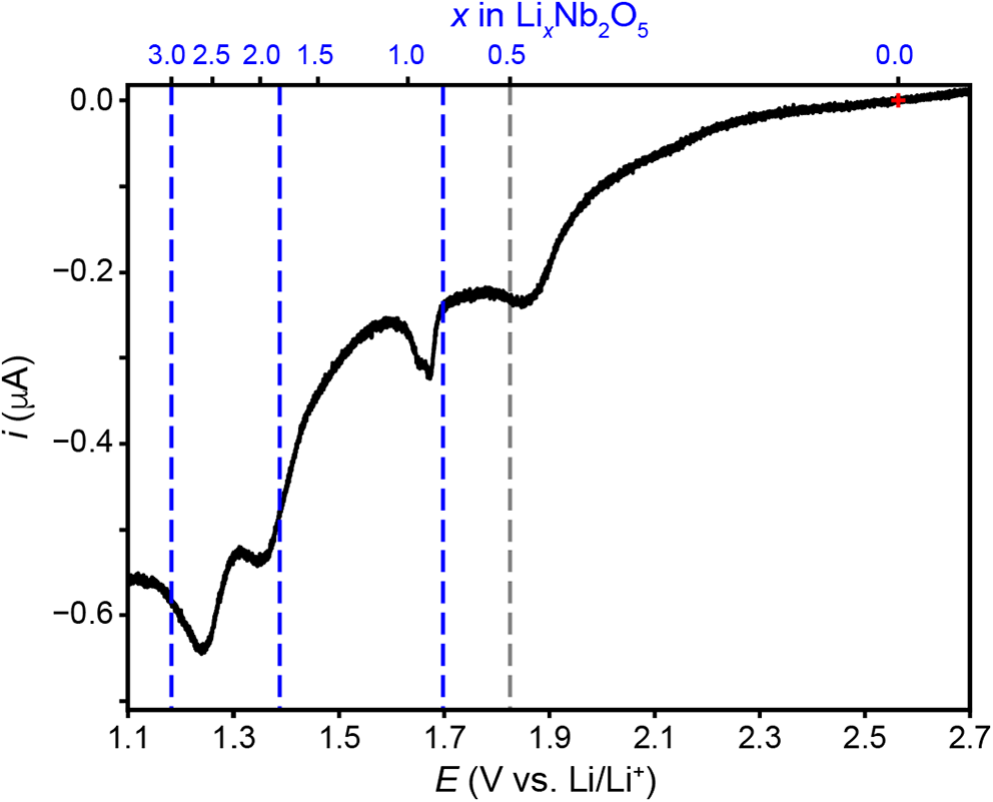
Linear sweep voltammogram of a T-Nb_2_O_5_ electrode at 1.5 μV/s scan rate. The black vertical line at *x* = 0.5 represents the DFT-predicted phase transition from (o) to (m) Nb_2_O_5_.^13^ The blue lines at *x* = 0.8, 1.8, and 3.0 represent experimentally determined phase transitions from orthorhombic to monoclinic, monoclinic to amorphous insulating phase, and amorphous insulating to a tetragonal phase, respectively.^13^

To reduce the total experiment time, we conducted an additional SSCV at a slightly faster scan rate of 2.5 μV/s. Notable changes emerged in the second scan (red trace in Figure 6). The sharp, reversible peaks at 1.678 and 1.658 V that were previously observed over more than 20 cycles (Figure 3) disappeared. Yang et al. also observed sharp cathodic peaks at 1.78 and 1.61 V during the first CV cycle (50 μV/s) for 100 nm sphere-like T-Nb_2_O_5_ particles.^29^ These peaks disappeared after subsequent cycling to 1.0 V, suggesting irreversible structural or compositional changes occur during the initial lithiation process. In addition, abrupt current fluctuations were detected, indicating intermittent electrical discontinuities in the cell, likely caused by mechanical damage to the electrode that resulted in temporary open-circuit behavior. Lastly, the cathodic peaks below 1.6 V were no longer observed.

## Discussion

We observed three broad cathodic peaks at 1.95, 1.35, and 1.24 V upon lithiating a pristine T*-*Nb_2_O_5_ particle-based electrode from 3.0 to 1.2 V at *v* = 1.5 μV/s. In addition, we observed 2 sharp peaks at 1.678 and 1.658 V that merge into a single “super peak” at 200 μV/s. This “super peak” is widely observed in the literature.^11–13,20,22,23,30^ The “super peak” shape can depend on the synthesis conditions, material morphology (e.g., thin film, microparticles, or nanostructures), scan rate conditions, and potential cycling window. In some cases, multiple cathodic peaks were observed within the potential window of 1.6 to 2.0 V.^20^

First, we discuss the multi-electron redox behavior of T-Nb_2_O_5_. To do so, we replot the data from Figure 6 and calculate *x* as a function of applied potential (Figure 7). We achieved *x* = 3.0 in T-Li_*x*_Nb_2_O_5_ from 3.0–1.2 V. Although values approaching *x* ≈ 3 are rarely reported in recent studies, they are not unprecedented. Early work by Ohzuku et al.^6^ directly observed *x* ≈ 3 in T-Nb_2_O_5_ electrodes prepared without conductive carbon, and more recent measurements by Han et al.^13^ reached *x* ≈ 2.8 at C/34 in electrodes synthesized by the same high-temperature method used here. While conductive carbon improves electrode conductivity, its intrinsic lithiation occurs below ∼0.3 V vs Li/Li^+^ and does not exhibit sharp redox features in the higher potential range explored here, indicating that the electrochemical behavior reported is intrinsic to T-Nb_2_O_5_.^31^ These observations—and our own data—indicate that multi-electron redox (i.e., more than one electron per Nb in the unit cell) is accessible under extremely slow cycling conditions. Consistent with this, our cells cycled at conventional C/3 rates reach only *x* ≈ 1.7 (Figure 2), highlighting that access to *x* ≈ 3 is strongly rate-dependent rather than anomalous.

A single redox reaction has been used to describe the voltammetry of T-Nb_2_O_5_ (T – Nb_2_O_5_ + *x*Li^+^ + *xe* ^–^↔T – Li_*x*_Nb_2_O_5_ for 0 < *x* < 2). However, a single redox reaction cannot explain the five distinct peaks in our SSCV experiments. Here we discuss the likely origin of the peaks observed in SSCV data of T-Nb_2_O_5_ based on recent *in situ* X-ray diffraction and transmission electron microscopy (TEM) data obtained upon lithiating single-crystalline T-Nb_2_O_5_ thin film electrodes,^13^ as well as polycrystalline T-Nb_2_O_5_ powder samples.

Based on previous *in situ* structural analyses by Han et al, we propose that a monoclinic (m) distortion (γ ≈ 94°) of the initial orthorhombic (o) T-Nb_2_O_5_ structure is responsible for the first reduction peak in Figure 7 (i.e., *i*_p,c1_ at 1.95 V).^13^ Han et al. assigned this transition upon lithiating T-Nb_2_O_5_ powder samples to *x* = 0.8 and observing a large shift of the (180) diffraction peak position and a concomitant disappearance of the (181) peak. However, under the SSCV conditions employed herein, the first reduction peak occurs at *x* = 0.5 (Figure 7). Interestingly, DFT calculations by the same authors predict that T-Nb_2_O_5_ will undergo the orthorhombic to monoclinic phase transition at *x* = 0.5, in agreement with our experimental result. One possible explanation for why the first reduction peak in Figure 7 appears at a lower value of *x* in our SSCV data is the significantly longer experiment duration; 333 h for a single lithiation cycle compared to 17 h in the in situ XRD experiment. The extended time scale allows more time for the sample to reach equilibrium, resulting in the peak appearing at more positive potentials (i.e., lower *x* values). Han et al. noted that both (m) and (o) phases are electrochemically active for Li-ion insertion and 25% of the sample remains in the initial (o)-T-Nb_2_O_5_ phase. Penner and co-workers reported that both lithiated and unlithiated domains exist in electrochemically deposited T-Nb_2_O_5_ films.^28^ Hence, we assign the first reduction peak of T-Nb_2_O_5_ at 1.95 V to the following redox reaction: (o)­T – Nb_2_O_5_ + 0.5Li^+^ + 0.5*e* ^–^↔(m)­T – Li_0.5_Nb_2_O_5_.

Next, we discuss the origin of the sharp peaks at 1.678 and 1.658 V in Figure 3b that occur at *x* = 0.9. Griffith et al. observed similar peaks in d*Q*/d*V* data (C/10 conditions) from T-Nb_2_O_5_ particles synthesized under identical solid-state conditions,^14^ but did not assign their origin. Interestingly, the same authors did not observe the sharp peaks in single-crystal thin film electrodes.^13^ The presence of the peaks in particle-based electrodes, but not in thin films, suggests that a surface-related process is responsible for their origin; the peaks appear in particle-based electrodes due to the high surface area of the electrode. Surface-related electrochemical processes such as the electrochemical reduction of Nb surface atoms and adsorption of Li-ions, a surface phase transition^24,25^ (such as a change in the coordination sphere of oxygens around Nb centers from NbO_6_ to NbO_7_ and NbO_8_ coordinations),^28^ or surface reactions with the electrolyte^9^ could all be responsible for these sharp peaks. Several observations support this assignment. The peaks exhibit narrow widths (22 mV), small peak-to-peak splittings (15 mV), and a linear relationship between peak current and *v* are all consistent with surface-related electrochemical processes and an attraction energy between the intercalation sites.^24^ To assess whether the charge associated with the sharp redox peaks in Figure 3b can be explained by a surface-related process, we performed a straightforward calculation with a gross simplification of the electrode morphology: the calculation assumes monodisperse, pseudospheres of T-Nb_2_O_5_ with uniform surface atom density and neglects porosity, facet variation, and electrolyte wetting effects. Using the sample mass (1.45 × 10^–4^ g), density (4.6 g cm^–3^), and an estimated Nb surface atom density of ∼7 atoms nm^–2^ derived from TEM images,^13^ we computed the number of surface atoms for particle diameters between 100 and 5000 nm. Dividing the total charge (1 × 10^16^ electrons) by the estimated number of surface atoms yields values consistent within an order of magnitude (Figure S3), indicating that the measured charge could plausibly arise from surface Nb sites. Despite the simplifications, this back-of-the-envelope analysis supports the assignment of the sharp voltammetric peaks to surface-related redox activity.

We note that sharp features in the differential electrochemical response have previously been reported for B–Nb_2_O_5_.^14^ In particular, Griffith et al. observed pronounced peaks in d*Q*/d*V* and concluded that only ∼10% of Nb sites are electrochemically reduced in phase-pure B–Nb_2_O_5_, suggesting that these features may be associated with a surface or near-surface redox process rather than bulk lithium intercalation. It is possible that the sharp peaks observed here for T-Nb_2_O_5_ originate from a minor B–Nb_2_O_5_ impurity phase or from structurally related near-surface environments. We cannot definitively exclude this contribution. Distinguishing between intrinsic T-Nb_2_O_5_ redox behavior and contributions from minor B–Nb_2_O_5_ domains represents an important open question and motivates future targeted structural and spectroelectrochemical investigations. It is also possible that at ultra-slow scan rates, Li ions may have sufficient time to relax toward thermodynamically preferred configurations, potentially including site redistribution or ordering; however, resolving such effects requires structural probes beyond the scope of cyclic voltammetry^32^ and remains an open question. The precise origin of these sharp cathodic features remains unresolved and represents an open avenue for future investigation.

Finally, we discuss the likely origin of the two reduction peaks at 1.35 and 1.24 V respectively, corresponding to approximately *x* = 2.0 and 2.5, respectively. Based on the *operando* PXRD data from Han et al.,^13^ we assign the peak at 1.35 V to the formation of an amorphous insulating phase and the peak at 1.24 V to the formation of a tetragonal (*t*) Li-rich layered rock salt structure, respectively. The amorphous phase exhibits high capacity at high rates,^19^ but is an irreversible structural transition for initially crystalline samples.^13^ The cathodic peaks observed between 1.0 and 1.4 V in the first sweep are attributed to phase transitions in T-Nb_2_O_5_ and disappear in subsequent cycles due to irreversible degradation induced upon cycling below ∼1.1 V.^6^

## Conclusion

We synthesized T-Nb_2_O_5_ via a high temperature solid–solid state reaction and conducted SSCV at 1.5 μV/s to investigate the fundamental electrochemical behavior of the material under near-equilibrium conditions. We achieved *x* = 3.0 in T-Li_*x*_Nb_2_O_5_ from 3.0–1.2 V and observed five distinct cathodic peaks that have yet to be observed, presumably because they were obscured by ohmic losses incurred during typical CV conditions. The first reduction peak at 1.95 V is assigned to an orthorhombic-to-monoclinic phase transition associated with Li insertion to *x* ≈ 0.5. Two sharp peaks at 1.678 and 1.658 V likely arise from a surface-related redox process, likely involving electrochemical reduction of Nb surface atoms and adsorption of Li-ions, a surface phase transition (either a change in the coordination sphere of oxygens around Nb centers from NbO_6_ to NbO_7_ and NbO_8_ coordinations, or involving impurity phases) or surface reactions with the electrolyte; the charge involved corresponds to only a small fraction (<2%) of surface Nb atoms. Finally, the broader peaks at 1.35 and 1.24 V can be attributed to the formation of an amorphous Li-rich phase and a subsequent tetragonal layered rock-salt structure, respectively. Together, these assignments show that the complex voltammetry of T-Nb_2_O_5_ reflects both bulk phase transitions and surface-confined electrochemical processes.

## Supplementary Material



## Notes

Rietveld refinement data and additional SSCV measurements (PDF)

## †

Department of Chemistry, University of Wisconsin-Madison, Madison, Wisconsin 53706, United States

## ‡

Department of Chemistry, University of Washington, 109 Bagley Hall, Box 351700, Seattle, Washington 98195-1700, United States

## Abbreviations

SSCV: slow scan cyclic voltammetry
