# Supplementary material for: Slow Scan Cyclic Voltammetry of Li-Ion Insertion in T‑Nb2O5 Reveals Hidden Peaks and Multi-Electron Redox
Source: ACS Electrochem. 2026 Mar 19;2(4):974–82. doi: 10.1021/acselectrochem.5c00533 (PMC13051442; doi:10.1021/acselectrochem.5c00533)
Supplement: Supplementary file 1 [file ec5c00533_si_001.pdf]

Supplementary Information for

Slow Scan Cyclic Voltammetry of Li-ion Insertion in T-Nb_2O_5 Reveals Hidden Peaks and Multi-Electron Redox

*Luke D. Salzer, Cami Christensen, Claire Gervais, Jacob D. Steeley, James R. Neilson, and Justin B. Sambur**

*jsambur@colostate.edu

Department of Chemistry, Colorado State University, Fort Collins, CO 80523, USA

Content

Pg. S2 - Table S1. Rietveld refinement results.
Pg. S2 – Figure S1. Additional SSCV data.
Pg. S3 – Figure S2. Additional SSCV data from two different cells.
Pg. S4 – Figure S3. Estimated number of electrons stored in the sample.
Pg. S5 – Figure S4. Selected SSCV data from Figure 3a of the main text.

Table S1. Rietveld refinement results of the PXRD data in Figure 1 of the main text.

Refinement model	Mass fraction of T- Nb_2O_5	Mass fraction of B-Nb_2O_5	R_{wp} (%)
**T-Nb_2O_5**	100%	n/a	10.739
**T-Nb_2O_5 & B-Nb_2O_5**	98.2%	1.8%	10.127

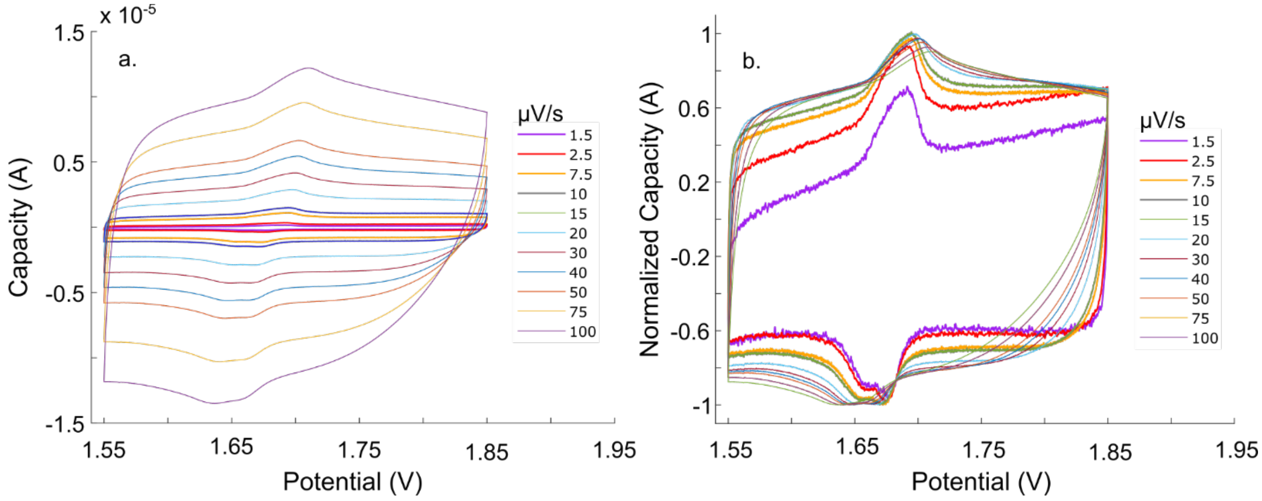

Figure S1. (a) SSCV of a T-Nb_2O_5 electrode that underwent 20 charge-discharge cycles from 3.0 to 1.2 V (b) Same data as in panel (a) normalized with respect to the maximum cathodic current.

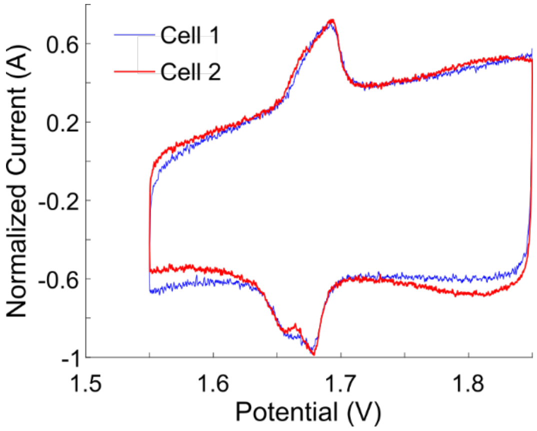

Figure S2. Comparison of SSCV data at at 1.5 μV/s for two different T-Nb_2O_5 electrodes that underwent 20 charge/discharge cycles from 3.0 V to 1.2 V in 1 M $LiClO_4$ in PC.

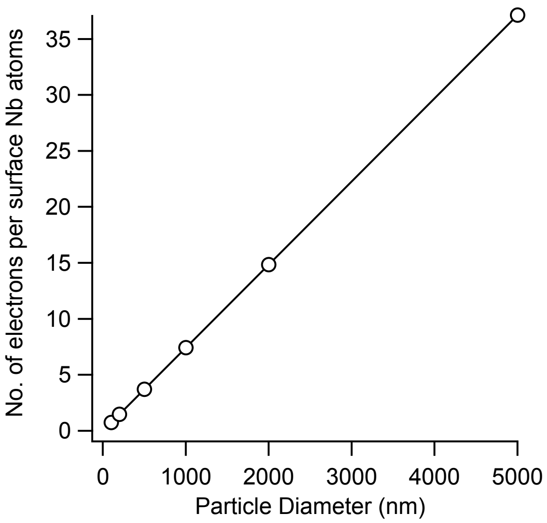

Figure S3. Estimated number of electrons stored in the T-Nb_2O_5 sample associated with $i_{p,c1}$ and $i_{p,c2}$ per number of Nb surface atoms. To calculate that charge-to-surface atom ration, we estimated the fraction of surface Nb atoms involved in charge storage by relating the total charge associated with the sharp peaks in Figure 3b (1.66×10^{8} moles electrons or 9.99×10^{15} electrons) to the estimated total number of surface atoms in our T-Nb_2O_5 samples. The total solid volume was calculated from the sample mass (1.45×10^{-4} g) and the density of T-Nb_2O_5 (4.6 g cm^{-3}). Assuming the particles are monodisperse, pseudospheres with diameters between 100 nm and 5000 nm, the number of particles was obtained by dividing the total solid volume by the volume of a single spherical particle. The total surface area was then determined by multiplying the number of particles by the surface area of a single sphere. The total number of surface Nb atoms was estimated by assuming a surface Nb areal density of 7 atoms nm^{-2}, consistent with the atomic packing density of Nb_2O_5 surfaces. Finally, the ratio of stored electrons to surface Nb atoms was calculated for each assumed particle size.

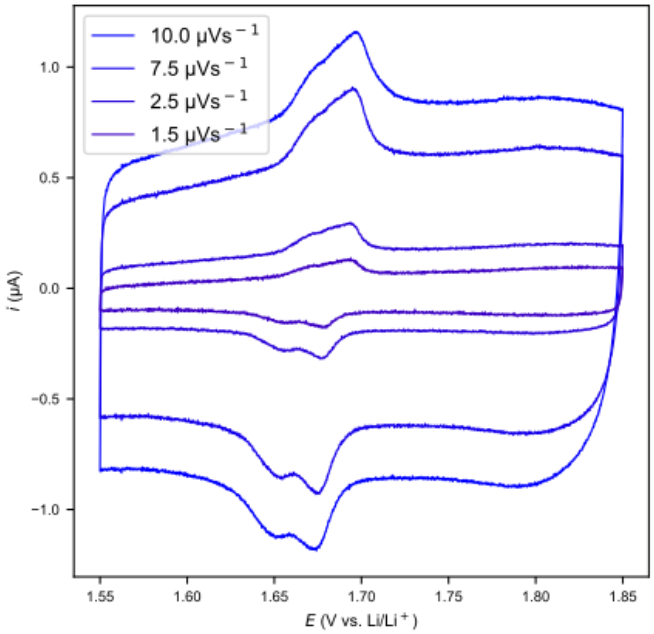

Figure S4. Selected SSCV data from Figure 3a of the main text, obtained using a T-Nb_2O_5 electrode that underwent 20 charge-discharge cycles from 3.0 to 1.2 V in 1M $LiClO_4$ in PC.
